# Nurr1 overexpression in the primary motor cortex alleviates motor dysfunction induced by intracerebral hemorrhage in the striatum in mice

**DOI:** 10.1016/j.neurot.2024.e00370

**Published:** 2024-05-03

**Authors:** Keita Kinoshita, Kensuke Motomura, Keisuke Ushida, Yuma Hirata, Ayumu Konno, Hirokazu Hirai, Shunsuke Kotani, Natsuko Hitora-Imamura, Yuki Kurauchi, Takahiro Seki, Hiroshi Katsuki

**Affiliations:** a Department of Chemico-Pharmacological Sciences, Graduate School of Pharmaceutical Sciences and School of Pharmacy, Kumamoto University, Kumamoto, Japan; b Department of Neurophysiology & Neural Repair, Gunma University Graduate School of Medicine, Gunma, Japan; c Global Center for Natural Resources Sciences, Graduate School of Pharmaceutical Sciences, Kumamoto University, Kumamoto, Japan; d Department of Pharmacology, School of Pharmacy, Himeji Dokkyo University, Hyogo, Japan

**Keywords:** Hemorrhagic stroke, Nurr1, Motor dysfunction, Corticospinal tract injury, Glial cell line-derived neurotrophic factor

## Abstract

Hemorrhage-induced injury of the corticospinal tract (CST) in the internal capsule (IC) causes severe neurological dysfunction in both human patients and rodent models of intracerebral hemorrhage (ICH). A nuclear receptor Nurr1 (NR4A2) is known to exert anti-inflammatory and neuroprotective effects in several neurological disorders. Previously we showed that Nurr1 ligands prevented CST injury and alleviated neurological deficits after ICH in mice. To prove direct effect of Nurr1 on CST integrity, we examined the effect of Nurr1 overexpression in neurons of the primary motor cortex on pathological consequences of ICH in mice. ICH was induced by intrastriatal injection of collagenase type VII, where hematoma invaded into IC. Neuron-specific overexpression of Nurr1 was induced by microinjection of synapsin I promoter-driven adeno-associated virus (AAV) vector into the primary motor cortex. Nurr1 overexpression significantly alleviated motor dysfunction but showed only modest effect on sensorimotor dysfunction after ICH. Nurr1 overexpression also preserved axonal structures in IC, while having no effect on hematoma-associated inflammatory events, oxidative stress, and neuronal death in the striatum after ICH. Immunostaining revealed that Nurr1 overexpression increased the expression of Ret tyrosine kinase and phosphorylation of Akt and ERK1/2 in neurons in the motor cortex. Moreover, administration of Nurr1 ligands 1,1-bis(3′-indolyl)-1-(*p*-chlorophenyl)methane or amodiaquine increased phosphorylation levels of Akt and ERK1/2 as well as expression of glial cell line-derived neurotrophic factor and Ret genes in the cerebral cortex. These results suggest that the therapeutic effect of Nurr1 on striatal ICH is attributable to the preservation of CST by acting on cortical neurons.

## Graphical abstract

**Figure:**
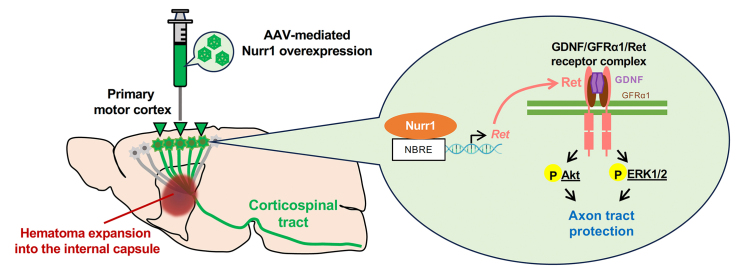

## Introduction

Intracerebral hemorrhage (ICH), triggered by rupture of blood vessels within brain parenchyma, is the second most frequent type of stroke. ICH is featured by high morbidity and mortality, inflicting 5 million people each year worldwide [1,2]. Although recent preclinical studies have been revealing the pathological mechanisms of ICH, effective therapeutic drugs for ICH are yet unavailable [3].

In humans, the putamen and the thalamus are particularly susceptible to ICH. When ICH occurs in these brain regions, hematoma often invades into the nearby internal capsule (IC), which contains axon bundles of the corticospinal tract (CST). Hemorrhage-induced CST injury in IC has been recognized to induce severe neurological dysfunction [4,5]. Aggravation of motor and sensorimotor deficits by invasion of hematoma into IC is also observed in experimental models of ICH induced in the striatum, where a blood-derived protease thrombin may mediate disruption of the structure and functions of axons [6,7]. In fact, several preclinical studies suggested that protection of CST contributed to the amelioration of ICH-induced neurological deficits [[8], [9], [10]]. Thus, CST protection is a potentially useful therapeutic approach for ICH.

Nurr1 (Nuclear receptor-related 1 protein or NR4A2), a member of nuclear receptor superfamily, plays essential roles in the development, maintenance, and regulation of dopaminergic neurons in the midbrain [11,12]. In addition, Nurr1 has been shown to exert anti-inflammatory functions by suppressing transcription of pro-inflammatory genes in microglia and astrocytes [13,14].

Although Nurr1 is recognized to possess ligand-independent constitutive activity as a transcription factor [15], several distinct series of compounds have been shown to bind to Nurr1 and modulate its transcription activities [[16], [17], [18]]. In this context, we have found that Nurr1 ligands may be useful as therapeutic agents for ICH. For example, intraperitoneal administration of amodiaquine (AQ), which binds to the ligand-binding domain of Nurr1 with high affinity, suppressed microglial activation in the peri-hematoma area, inhibited gene expression of inflammatory mediators, ameliorated CST injury and improved motor dysfunction after ICH in mice [19,20]. Moreover, oral administration of 1,1-bis(3′-indolyl)-1-(*p*-chlorophenyl)methane (C-DIM12), a Nurr1 ligand which binds to the co-activator site of Nurr1, also suppressed inflammatory responses, ameliorated CST injury and reduced motor dysfunction after ICH [19]. On the other hand, intraperitoneal administration of hydroxychloroquine, which shares common structures with AQ and binds to the ligand-binding domain of Nurr1, prevented CST injury and alleviated motor dysfunction but showed only limited effect on inflammatory responses [21]. These results implicated that Nurr1 and its ligands may produce therapeutic effects primarily via preservation of CST integrity.

Because CST originates from neurons in the primary motor cortex, here we carried out behavioral and histopathological examinations to reveal the effects of Nurr1 overexpression in these neurons on pathological consequences of ICH induced in the striatum in mice. We also examined the effects of Nurr1 ligands C-DIM12 and AQ on signaling events in the primary motor cortex to reveal potential mechanisms by which Nurr1 protects CST from ICH-induced injury.

## Materials and methods

### Ethics approval

All procedures were approved by the Animal Care and Use Committee of Kumamoto University (Approval numbers: A2022–006 and A2023-143), and animals were treated in accordance with U.S. National Research Council's Guide for the Care and Use of Laboratory Animals. Experiments using adeno-associated virus (AAV) vectors were approved by the Kumamoto University Ethics Committee concerning experiments on genetically modified organisms (Approval number: 5–016) and were performed under the Cartagena Protocol on Biosafety to the Convention on Biological Diversity.

### Adeno-associated virus vectors

**Fig. 1:**
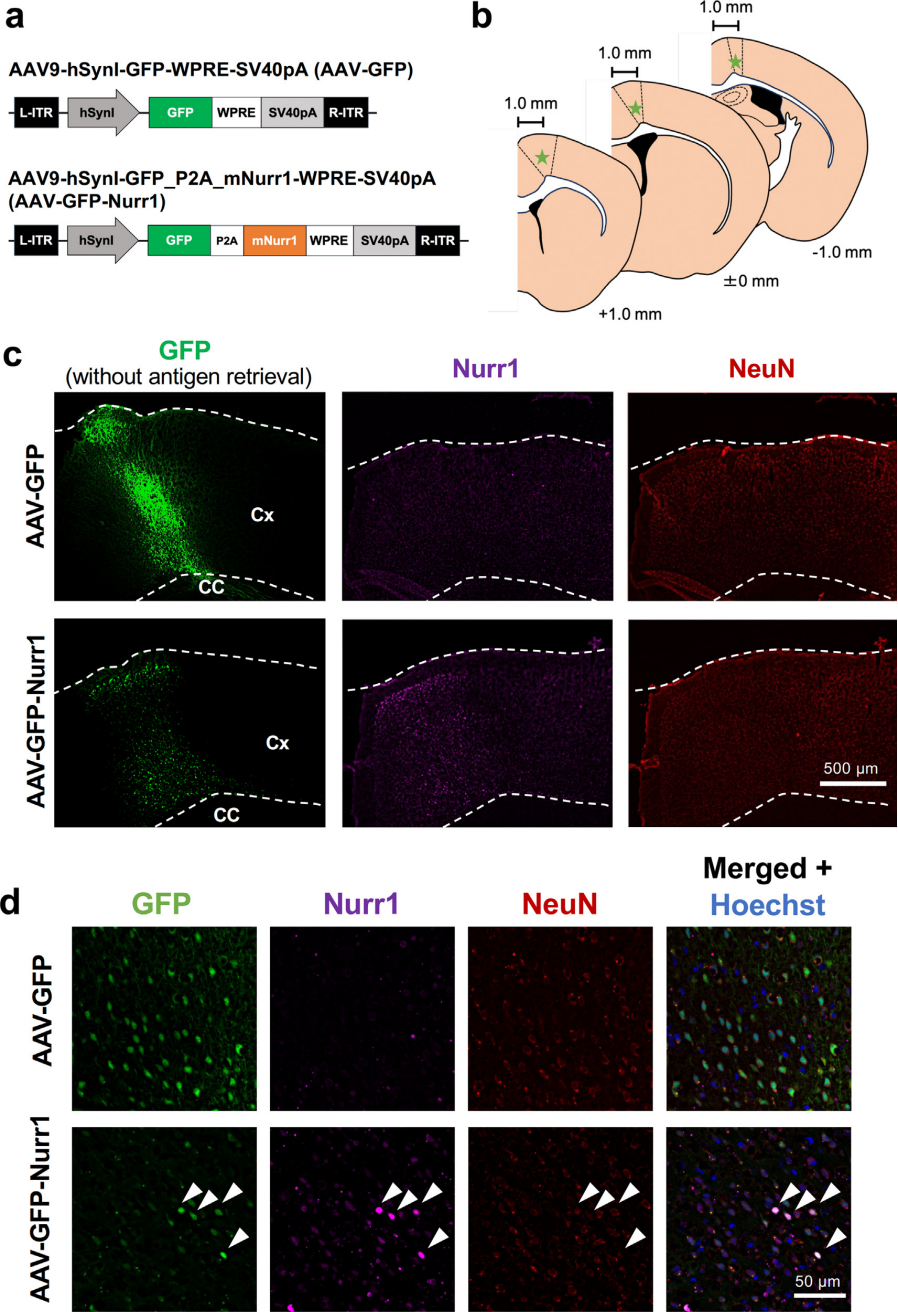
Confirmation of AAV vector-mediated GFP expression and Nurr1 overexpression *in vitro* and *in vivo.***a** Constructions of AAV vectors for introduction of GFP gene as control (top) and for introduction of GFP and Nurr1 genes (bottom). Neuron-specific gene expression was driven by human synapsin I (hSynI) promoter. **b** Schematic representation of the locations (indicated by stars) of stereotaxic injections of AAV vectors in the primary motor cortex (delineated by broken lines). **c** Representative low-magnification images of GFP fluorescence and Nurr1/NeuN immunofluorescence of cortical sections from mice at 4 weeks after AAV vector injection. The middle and right images are of the same sections, while the left image was derived from an adjacent section without antigen retrieval to clearly detect GFP fluorescence. Cx: cerebral cortex, CC: corpus callosum. **d** Magnified views of the regions expressing GFP in the primary motor cortex. Images of nuclear counterstaining with Hoechst 33342 are also shown. Arrowheads indicate merged cells.

We used AAV serotype 9 (AAV9) vector to express green fluorescent protein (GFP) and mouse Nurr1 (mNurr1) under neuron-specific human synapsin I (hSynI) promotor. Construction of the AAV expression plasmid was based on procedures described in previous studies [22,23]. First, the mNurr1 cDNA plasmid (Clone ID: K230026C20 (Nr4a2), DANAFORM, Yokohama, Japan) was cut with NotI, then amplified by polymerase chain reaction (PCR) and extracted. DNA fragments were amplified again using primers with EcoRI and NotI cleavage sites and extracted. The extracted fragment was inserted into AAV expression plasmid cleaved with EcoRI and NotI using In-Fusion Snap Assembly Master Mix (Takara Bio Inc., Shiga, Japan) to generate pAAV-hSynI-GFP_P2A_mNurr1-WPRE-sv40pA. pAAV9-hSynI-GFP-WPRE-sv40pA was used as the control vector (Fig. 1a). AAV9 vectors were produced using the ultracentrifugation method described in a previous paper [24].

### Primary cerebellar cultures

Rat primary cerebellar cultures were prepared as previously described [23,25]. Briefly, the cerebellums of embryonic day 16 embryos were obtained from pregnant Wistar/ST rats (Japan SLC Inc., Shizuoka, Japan) with the use of Neuron Dissociation Solutions (FUJIFILM Wako Chemicals, Osaka, Japan). Dissociated cells were cultured in Neuron Culture Medium (FUJIFILM Wako Chemicals) supplemented with 100 pM 3,3′,5′-triiodo-l-thyronine for 21 ​d *in vitro* (DIV21). AAV vectors (multiplicity of infection of 100,000) were added to primary cerebellar cultures on DIV7 to express mNurr1. The cells were fixed with 4% paraformaldehyde (PFA) on DIV21.

### Injection of AAV vectors into the mouse cortex

Male ICR mice (Slc:ICR; Nihon SLC, Shizuoka, Japan) at 4 weeks of age were maintained at constant ambient temperature (22 ​± ​1 ​°C) under a 12-h light/dark cycle with food and water available ad libitum. For the surgery, mice were placed in a stereotaxic frame after anesthesia with an intraperitoneal injection of the combination anesthetic consisting of 0.3 ​mg/kg medetomidine, 4.0 ​mg/kg midazolam and 5.0 ​mg/kg butorphanol. A 30-gauge needle was inserted into three sites of the primary motor cortex (stereotaxic coordinates; 1.0 ​mm lateral to the midline, 1.0, 0, −1.0 ​mm anterior to the bregma, and 0.8 ​mm deep below the brain surface). AAV9 vector solution (5.0 ​× ​10^12^ vg/mL) in phosphate-buffered saline (PBS) were injected in a volume of 0.2 ​μL per site at a constant rate of 0.2 ​μL/min with the use of a microinfusion pump (Fig. 1b).

### ICH mouse model

ICH was induced by intrastriatal injection of collagenase in male ICR mice at 8 weeks of age, as described previously [26,27]. Mice were placed in a stereotaxic frame after anesthesia by the combination anesthetic. A 30-gauge needle was inserted into the striatum (stereotaxic coordinates; 2.3 ​mm lateral to the midline, 0.2 ​mm anterior to the bregma, and 3.5 ​mm deep below the skull) to inject 0.035 U collagenase type VII (Sigma-Aldrich Japan, Tokyo, Japan) in 0.5 ​μL of physiological saline, at a constant rate of 0.2 ​μL/min with a microinfusion pump. The needle remained in place for over 5 ​min and then was slowly withdrawn.

### Assessments of motor/sensorimotor functions

Motor and sensorimotor functions of mice were evaluated by the beam-walking test and the modified limb-placing test, respectively, by investigators blinded to the treatments at 0, 6, 24, 72 and 168 ​h after ICH induction. In the beam-walking test, mice were trained before surgery to walk on a beam with 15 ​mm width, 1.1 ​m length and 0.5 ​m height. The fault rate means the rate of hindlimb foot slips during crossing the beam. The distance score means the number of 0.1-m sections across the beam (11 sections in total) that the mouse could reach from an end without falling. The performance score of mice was based on a seven-point scale as previously described [19,28]. These parameters were obtained as the average value from three trials at each time point. In the modified limb-placing test [19,28], mouse was suspended 0.1 ​m over a table, and the stretch of the forelimbs toward the table was observed and evaluated. Next, the mouse was positioned along the edge of the table with its forelimbs suspended over the edge and allowed to move freely. Each limb was pulled down gently, and retrieval and placement were checked. Finally, the mouse was placed toward the table edge to check for lateral placement of the forelimb. A total of seven points means maximal neurological deficits, and a total of 0 points means normal performance.

### Immunofluorescence

Mice were deeply anesthetized again and perfused transcardially with 30 ​mL of ice-cold PBS followed by 30 ​mL of 4% PFA. Brains were isolated and post-fixed in 4% PFA overnight at 4 ​°C, and then soaked in 15% sucrose overnight at 4 ​°C. After freezing, they were cut into sections of 16 ​μm thickness, and three sections around the injection site were collected and stored at −80 ​°C. Antigen retrieval was performed by incubation in 10 ​mM citrate buffer (pH 8.0–8.5) for 30 ​min at 85 ​°C. After rinsing with PBS containing 0.3% Triton X-100 (PBS/T), specimens were treated with PBS/T containing blocking serum for 1 ​h at room temperature, then incubated with primary antibodies overnight at 4 ​°C. Primary antibodies were rabbit anti-Nurr1/NR4A2 polyclonal antibody (1:100 or 200; RRID:AB_2153760, Proteintech, Rosemont, IL, USA), mouse anti-NeuN monoclonal antibody (1:500; RRID:AB_2313673, Millipore Corporation, Billerica, MA, USA), mouse anti-Neurofilament-H (1:500; RRID:AB_10694081, Cell Signaling Technology Japan, Tokyo, Japan), rabbit anti-Iba1 antibody (1:500; RRID:AB_839504, FUJIFILM Wako Chemicals), mouse monoclonal anti-glial fibrillary acidic protein (GFAP) antibody (1:500; RRID:AB_11212597, Millipore Corporation), rabbit anti-nitrotyrosine (1:500; RRID:AB_310089, Millipore Corporation), rabbit anti-beta-Amyloid Precursor Protein (APP, 1:100; RRID:AB_2533275, Thermo Fisher Scientific K.K., Tokyo, Japan), rabbit anti-Ret (C31B4) monoclonal antibody (1:100; RRID:AB_2238465, Cell Signaling Technology), rabbit anti-phospho-Akt (Ser473) (1:100; RRID:AB_329825, Cell Signaling Technology), and rabbit anti-phospho-p44/42 MAPK (ERK1/2) (Thr202/Tyr204) (1 : 200; RRID:AB_331646, Cell Signaling Technology). After rinsing with PBS/T, specimens were incubated with corresponding secondary antibodies for 2 ​h at room temperature. Secondary antibodies were Alexa Fluor 555 donkey anti-rabbit IgG (H ​+ ​L) antibody (1:500; RRID:AB_162543, Thermo Fisher Scientific K.K.), Alexa Fluor 647 donkey anti-rabbit IgG (H ​+ ​L) antibody (1:500; RRID:AB_2536183, Thermo Fisher Scientific K.K.), Alexa Fluor 488 donkey anti-mouse IgG (H ​+ ​L) antibody (1:500; RRID:AB_141607, Thermo Fisher Scientific K.K.) and Alexa Fluor 555 donkey anti-mouse IgG (H ​+ ​L) antibody (1:500; RRID:AB_2536180, Thermo Fisher Scientific K.K.). Fluorescence signals were observed with the use of THUNDER microscope (Leica Microsystems Inc., IL, USA). Axonal shape index was calculated as a ratio of the length and width of individual GFP-positive or neurofilament-H-positive signals with the use of NIH ImageJ software (RRID:SCR_003070, National Institutes of Health, Bethesda, MD, USA) based on the procedures reported in a previous study [7]. Ten GFP-positive or fifteen neurofilament-H-positive fibers in an image of 120 ​× ​120 ​μm^2^ were randomly selected for the measurement, and the averaged percentage from two or three sections was taken as the value for each mouse by an investigator blinded to the conditions of AAV injections. The number of Iba1-immunopositive cells per 270 ​× ​360 ​μm^2^ in the peri-hematoma region was counted by an investigator blinded to the conditions of AAV injections [20]. The average number of cells from three sections was taken as the value for each mouse. For GFAP immunoreactivity, threshold-based quantification of the immunopositive area was conducted with NIH ImageJ software. The percentage of GFAP-immunopositive area of 270 ​× ​360 ​μm^2^ in the peri-hematoma region was obtained from each section, and the averaged percentage from three sections was taken as the value for each mouse. Nitrotyrosine-positive area was quantified with the use of NIH ImageJ software, and the averaged percentage from three sections was taken as the value for each mouse. Quantification of NeuN-immunopositive cells in the center of hematoma was performed similarly as Iba1-immunopositive cells. Threshold-based quantification of APP-immunopositive area (%) was conducted with NIH ImageJ software in an image of 270 ​× ​360 ​μm^2^, and the average percentage from three sections was taken as the value for each mouse. The percentage of Ret-, phospho-Akt (pAkt)- and phosphor-ERK1/2 (pERK1/2)-positive cells in GFP positive cells was obtained from an area of 211 ​× ​211 ​μm^2^ in the primary motor cortex, and the averaged percentage from more than three sections was taken as the value for each mouse.

### Estimation of injury volume

Procedures for estimation of the injury volume were essentially based on those reported in our previous study [20]. Coronal brain sections of 16 ​μm thickness obtained every 208 ​μm were subjected to Nissl staining to visualize the region injured by hematoma. Injured area in each section spanning the entire hematoma was measured by NIH ImageJ software. Injury volume of individual mice was determined by integration of the injured area in each section over the section depth.

### Real-time quantitative polymerase chain reaction (RT-qPCR)

At 1 ​d after ICH induction, mice were deeply anesthetized again and transcardially perfused with 30 ​mL ice-cold PBS. Brain tissues more than 2.0 ​mm dorsal from bregma were removed, and only the cerebral cortex was isolated by tweezers. The cortical sample was stored in 2 ​mL RNAiso Plus reagent (Takara Bio Inc.), and total RNA was extracted. Reverse transcription of total RNA into cDNA was performed under the conditions of 1 cycle at 37 ​°C for 15 ​min and 85 ​°C for 5 ​s, using Prime Script™ RT Master Mix (Takara Bio Inc.). Obtained cDNA solution was subjected to real-time PCR (a cycle at 95 ​°C for 30 ​s, 40 cycles at 95 ​°C for 15 ​s, 55 ​°C for 45 ​s, and 72 ​°C for 30 ​s) in CFX Connect™ Real-time System (Bio-Rad Laboratories, Inc. Hercules, CA, USA) with the use of KAPA SYBR Fast qPCR kit (Japan Genetics Inc., Tokyo, Japan). Glyceraldehyde-3-phosphate dehydrogenase (GAPDH) was used as the internal control. The primer sequences were as follows: *Ret* forward, 5′- TCAGTACACGGTGGTAGCCACT -3’; *Ret* reverse, 5′-CGCCTCTTGTTTACTGCACAGG-3’; *Gdnf* forward, 5′-CCTTCGCGCTGACCAGTGACT-3’; *Gdnf* reverse, 5′-GCCGCTTGTTTATCTGGTGACC-3’; *Gfrα1* forward, 5′- AGACTTCCACGCCAACTGTCGA-3’; *Gfrα1* reverse, 5′- GGAGTCCACATAGTTCGGTGTC-3’; *Gapdh* forward, 5′-ACCATCTTCCAGGAGCGAGA-3’; *Gapdh* reverse, 5′-CAGTCTTCTGGGTGGCAGTG-3’. Data were automatically analyzed with a comparative Ct method.

### Western blotting

At 3 ​d after ICH induction, mice were deeply anesthetized again and transcardially perfused with 30 ​mL ice-cold PBS. The cortical sample was isolated by the same procedures as those for RT-qPCR and was homogenized in 1 ​mL ice-cold lysis buffer (150 ​mM NaCl, 50 ​mM Tris-HCl (pH 7.5), 5 ​mM EDTA, 1% Nonidet P-40, 0.1% sodium dodecyl sulfate (SDS), 0.5% sodium deoxycholate, 0.1% sodium orthovanadate, 50 ​mM sodium fluoride and 1% protease inhibitor cocktail). After incubation on ice for 30 ​min, lysates were centrifuged at 14,000 ​rpm at 4 ​°C for 30 ​min, and the protein concentration in each sample was determined by the bicinchoninate method. With added sample buffer containing 0.5 ​M Tris-HCl (pH 6.8), 10% SDS, 2-mercaptoethanol, glycerol and 1% bromophenol blue, each sample was heated at 100 ​°C for 10 ​min. SDS polyacrylamide gel electrophoresis was performed on a 5.4% stacking gel with 10% separating gel. After gel electrophoresis, proteins were transferred onto polyvinylidene difluoride membranes. The blots were washed with Tris-buffered saline containing 0.1% Tween 20 and blocked with Blocking One (Nacalai Tesque, Kyoto Japan) at room temperature for 60 ​min. The membrane was incubated with rabbit anti-phospho-Akt (Ser473) (1 : 1000), rabbit anti-Akt (1 : 1000; RRID:AB_329827, Cell Signaling Technology), rabbit anti-phospho-p44/42 MAPK (ERK1/2) (Thr202/Tyr204) (1:1000), rabbit anti-p44/42 MAPK (ERK1/2) (1:1000; RRID:AB_390779, Cell Signaling Technology), and mouse anti-β-actin antibody (1:1000; RRID:AB_476744, Sigma-Aldrich Japan) overnight at 4 ​°C. After incubation with horseradish peroxidase-conjugated secondary antibodies at room temperature for 1 ​h, bands were detected with Clarity™ Western ECL Substrate (ThermoFisher Scientific K.K.) on a luminoimaging analyzer (FUSION SOLO; Vilber-Lourmat, France) and analyzed by NIH ImageJ software.

### Drug administration

C-DIM12 was synthesized as described previously and administered to mice at 50 ​mg/kg/d by oral gavage [19]. AQ was obtained from Cypex Ltd. (Dundee, UK) and administered intraperitoneally to mice at 40 ​mg/kg/d as reported previously [20]. In RT-qPCR experiments, these drugs were administered at 3 ​h after induction of ICH, and the cortical tissues were harvested at 1 ​d after ICH induction. In Western blot experiments, drugs were administered at 3 ​h after induction of ICH, then daily at 24-h intervals. Cortical tissues for western blotting were harvested at 3 ​d after ICH induction.

### Statistical analysis

All data are presented as mean ​± ​S.E.M. unless otherwise specified. Data on body weight were analyzed by two-way analysis of variance followed by post hoc comparisons with Sidak's method. Behavioral data were analyzed by two-way analysis of variance followed by post hoc comparisons with Bonferroni's method. In experiments including two group comparisons, data were analyzed by Student's *t*-test. When data sets included more than two groups, data were analyzed by one-way analysis of variance followed by Tukey's multiple comparisons test or analyzed by non-parametrical Kruskal-Wallis test followed by Dunn's multiple comparisons test. Statistical analysis was carried out with the GraphPad Prism software (RRID:SCR_002798, Graph Pad, San Diego, CA, USA). Two-tailed probability values ​< ​5% were considered significant.

## Results

### AAV vector-mediated Nurr1 overexpression in cortical neurons

We constructed an AAV vector expressing GFP and mNurr1 under the control of the neuron-specific hSynI promoter (hereafter, AAV-GFP-Nurr1). GFP and mNurr1 were independently expressed through the insertion of a self-cleaving sequence of the 2A peptide derived from porcine teshovirus-1 (P2A) [29]. As a control vector, the AAV vector expressing only GFP driven by hSynI promoter (hereafter, AAV-GFP) was used (Fig. 1a). To confirm induction of GFP and Nurr1 *in vitro*, primary cultured rat cerebellar cells were infected with these AAV vectors. Expression of GFP was induced by both AAV vectors at 14 ​d after infection. In addition, Nurr1-immunopositive signals were clearly observed in cerebellar cultures infected with AAV-GFP-Nurr1 but not in cultures infected with AAV-GFP (Supplementary Fig. 1). After validation of AAV vectors *in vitro*, we injected these vectors into the primary motor cortex of mice (Fig. 1b). At 4 weeks after AAV injections, both vectors induced GFP expression, whereas only AAV-GFP-Nurr1 vector induced enhanced expression of Nurr1 in GFP expressing area of serial brain sections (Fig. 1c). Magnified views of the regions positive for GFP expression in the primary motor cortex exhibited colocalization of Nurr1-and NeuN-immunopositive signals in GFP-expressing cells in mice injected AAV-GFP-Nurr1 vector (Fig. 1d).

### Nurr1 overexpression in the primary motor cortex ameliorated motor dysfunction after ICH

**Fig. 2:**
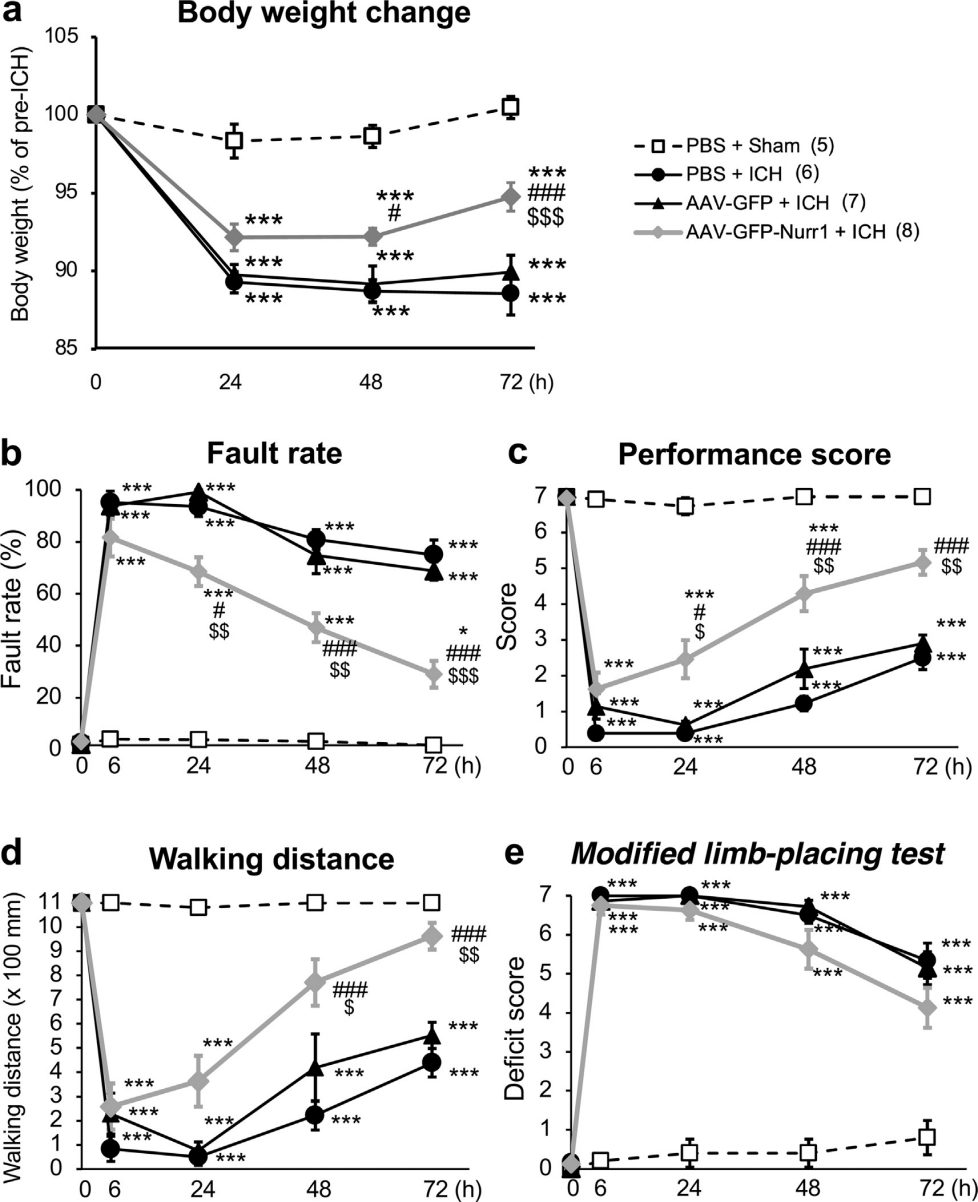
Effect of Nurr1 overexpression on body weight change and motor dysfunction after ICH induction. **a** Time-dependent changes in body weight of mice after sham operation or ICH induction. Significant differences between groups were observed by two-way repeated measure ANOVA (interactions, *F*_9, 66_ ​= ​11.63, *P* ​< ​0.001; time, *F*_3, 66_ ​= ​121.9, *P* ​< ​0.001; treatment, *F*_3, 22_ ​= ​25.84, *P* ​< ​0.001). **b-d** Results in the beam-walking test were evaluated by fault rate (**b**), performance score (**c**) and walking distance (**d**). Significant differences between groups were observed by two-way repeated measure ANOVA concerning the fault rate (interactions, *F*_12, 88_ ​= ​19.21, *P* ​< ​0.001; time, *F*_4, 88_ ​= ​167.4, *P* ​< ​0.001; treatment, *F*_3, 22_ ​= ​90.38, *P* ​< ​0.001), the performance score (interactions, *F*_12, 88_ ​= ​14.10, *P* ​< ​0.001; time, *F*_4, 88_ ​= ​125.8, *P* ​< ​0.001; treatment, *F*_3, 22_ ​= ​90.47, *P* ​< ​0.001) and the walking distance (interactions, *F*_12, 88_ ​= ​8.146, *P* ​< ​0.001; time, *F*_4, 88_ ​= ​64.23, *P* ​< ​0.001; treatment, *F*_3, 22_ ​= ​56.00, *P* ​< ​0.001). **e** Results in the modified limb-placing test. Significant differences between two groups were observed by two-way repeated measure ANOVA (interactions, *F*_12, 88_ ​= ​17.82, *P* ​< ​0.001; time, *F*_4, 88_ ​= ​203.1, *P* ​< ​0.001; treatment, *F*_3, 22_ ​= ​145.7, *P* ​< ​0.001). *∗P* ​< ​0.05 and *∗∗∗P* ​< ​0.001 versus PBS ​+ ​sham; *#P* ​< ​0.05 and *###P* ​< ​0.001 versus PBS ​+ ​ICH; *$ P* ​< ​0.05, *$$ P* ​< ​0.01 and *$$$ P* ​< ​0.001 versus AAV-GFP ​+ ​ICH. Post hoc test was based on Sidak's multiple comparison test (**a**) or Bonferroni's method (**b-e**). Number of mice examined was 5 in PBS ​+ ​sham group, 6 in PBS ​+ ​ICH group, 7 in AAV-GFP ​+ ​ICH group and 8 in AAV-GFP-Nurr1 + ICH group, respectively.

We next examined the effect of Nurr1 overexpression on body weight and motor functions of mice. During the post-injection period of 4 weeks after injection of vehicle or AAV vectors into the primary motor cortex, we observed no significant differences among three experimental groups (PBS, AAV-GFP and AAV-GFP-Nurr1) with regard to body weight and various parameters of neurological functions (Supplementary Fig. 2). In other words, AAV-mediated GFP expression and Nurr1 overexpression did not affect motor and sensorimotor functions of mice. We next induced ICH in the striatum at 4 weeks after AAV injection and addressed the effect of Nurr1 overexpression on ICH-induced body weight loss and neurological dysfunction. Significant decreases in body weight were observed in mice that suffered from ICH as compared to those with sham operation, but mice injected with AAV-GFP-Nurr1 showed significant recovery of body weight at 72 ​h after ICH induction (Fig. 2a). In the beam-walking test, mice that received PBS injection or AAV-GFP injection exhibited severe motor dysfunction as reflected by increases in the fault rate, decreases in the performance score and decreases in the walking distance. As compared to these groups of mice, mice injected with AAV-GFP-Nurr1 showed significantly better performance in these parameters (Fig. 2b–d). We also evaluated neurological symptoms of mice by the modified limb-placing test that reflected both sensory and motor functions. In this test, Nurr1 overexpression in the primary motor cortex by AAV-GFP-Nurr1 produced only modest effect on ICH-induced deficits, as compared to PBS injection and AAV-GFP injection (Fig. 2e).

### Nurr1 overexpression in the primary motor cortex prevents ICH-induced CST injury but does not affect other neuropathological events associated with hematoma

**Fig. 3:**
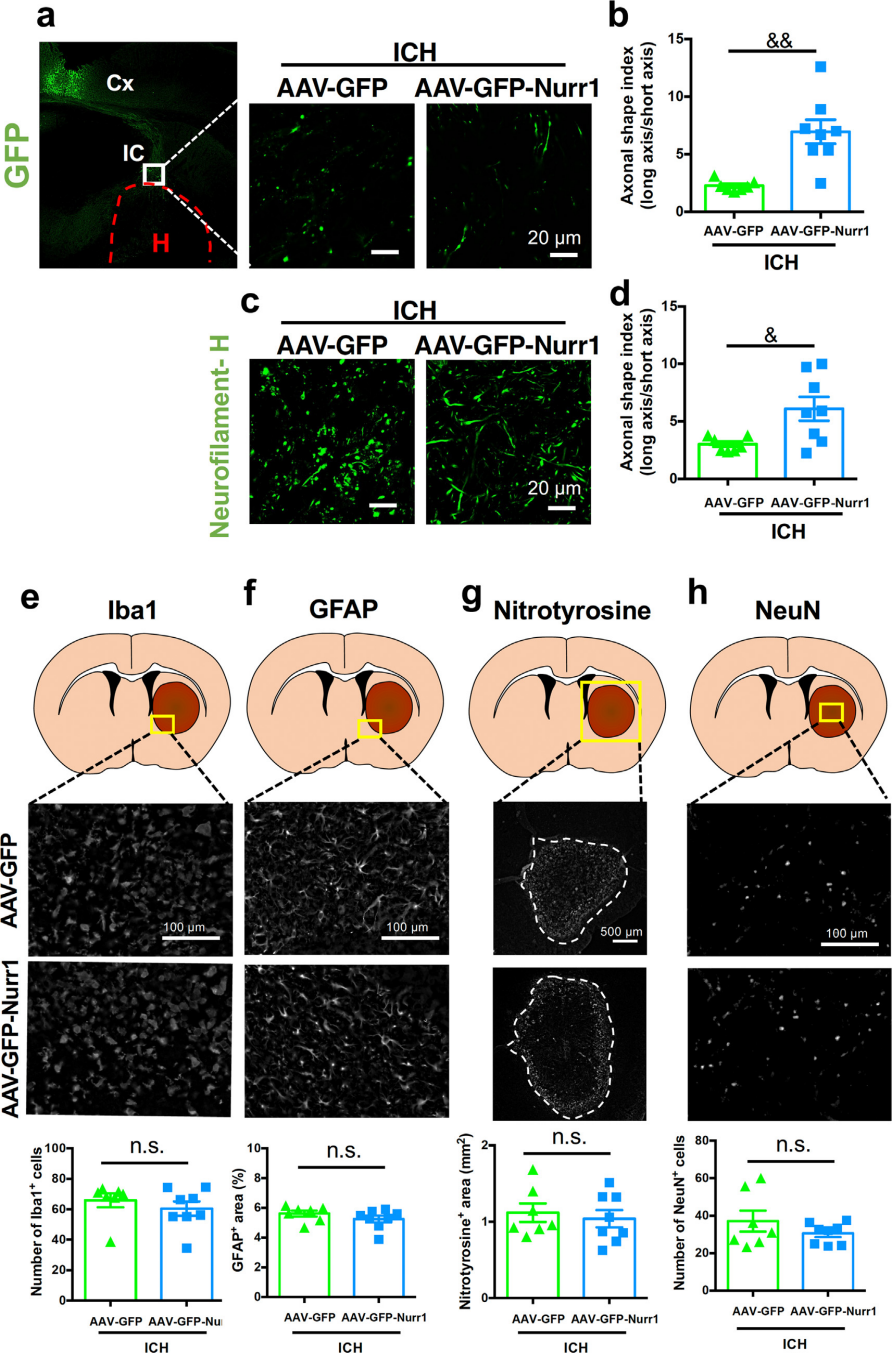
Effect of Nurr1 overexpression on neuropathological changes in the internal capsule and the striatum after ICH induction. **a** Left, a low magnification image of GFP fluorescence at 4 weeks after AAV-GFP injection. Cx: cerebral cortex, IC: internal capsule, H: hematoma. Middle and right, representative images of GFP fluorescence in the internal capsule at 3 ​d after ICH induction. **b** Quantitative results of morphological changes of GFP-positive axonal fibers after ICH induction. *$$ P* ​< ​0.01. **c** Representative images of neurofilament-H immunofluorescence in the internal capsule at 3 ​d after ICH induction. **d** Quantitative results of morphological changes of neurofilament-H-immunopositive axonal fibers after ICH induction. *$ P* ​< ​0.05. **e** Top, schematic representation of the area examined for Iba1 immunofluorescence. Middle, representative images of Iba1-immunopositive signals in peri-hematoma region at 3 ​d after ICH induction. Bottom, quantitative results on the number of Iba1-positive cells. **f** Top, schematic representation of the area examined for GFAP immunofluorescence. Middle, representative images of GFAP-immunopositive signals in peri-hematoma region at 3 ​d after ICH induction. Bottom, quantitative results on GFAP-positive area. **g** Top, schematic representation of the area examined for nitrotyrosine immunofluorescence. Middle, representative images of nitrotyrosine-immunopositive signals at 3 ​d after ICH induction. Broken lines indicate the edge of hematoma. Bottom, quantitative results on nitrotyrosine-positive area. **h** Top, schematic representation of the area examined for NeuN immunofluorescence. Middle, representative images of NeuN-immunopositive signals in the central region of the hematoma for at 3 ​d after ICH induction. Bottom, quantitative results on the number of NeuN-positive cells. n.s., not significant. Data were statistically analyzed by unpaired *t*-test. Number of mice examined was 7 in AAV-GFP ​+ ​ICH group and 8 in AAV-GFP-Nurr1 ​+ ​ICH group, respectively.

Because AAV-mediated Nurr1 overexpression significantly ameliorated motor dysfunction after ICH induction, we next examined the effect of Nurr1 overexpression on axonal structures in IC that contained axonal fiber bundles originated from the primary motor cortex. Axons of cortical neurons infected with AAV vectors were identified by GFP fluorescence, and the integrity of axonal structures was quantified as axonal shape index [7]. In our previous studies we showed that axonal shape index reflected the structural integrity of axon bundles in the internal capsule [7] and that several drugs effective in preventing the decrease of axonal shape index alleviated motor deficits associated with ICH [10,19,21]. GFP-positive axons in IC of mice infected with AAV-GFP exhibited severe degeneration revealed by their fragmented appearance at 3 ​d after induction of ICH. In contrast, axon structures of neurons infected with AAV-GFP-Nurr1 retained fibrous appearance, which was reflected by a significant increase in axonal shape index as compared to those infected with AAV-GFP (Fig. 3a and b). We also performed neurofilament-H immunostaining to evaluate overall integrity of axon structures in the internal capsule at 3 ​d after induction of ICH. Again, a significant ameliorating effect of Nurr1 overexpression on axonal shape index was observed (Fig. 3c and d). The extent of amelioration observed by neurofilament-H-based analysis was smaller than that observed by GFP-based analysis, which may be attributable to the fact that the former analysis included axons that did not express exogenous Nurr1 sufficiently.

Induction of ICH in the striatum produces various prominent neuropathological changes within and around hematoma, which include activation of microglia/macrophages and accumulation of activated astrocytes in the peri-hematoma region [30], upregulation of pro-inflammatory factors such as inducible nitric oxide synthase (iNOS) [31,32], and neuronal death within the hematoma [33]. We have previously reported that these neuropathological events could be reduced by systemic administration of Nurr1 ligands [[19], [20], [21]]. Accordingly, we addressed whether AAV-mediated Nurr1 overexpression in the primary motor cortex somehow affected general neuropathological events associated with hematoma produced in the striatum. Nurr1 overexpression in the primary motor cortex did not affect activation of Iba1-positive microglia/macrophages in the peri-hematoma region (Fig. 3e), accumulation of GFAP-positive astrocytes in the peri-hematoma region (Fig. 3f), increases in nitrotyrosine immunoreactivity reflecting nitric oxide-mediated nitrosative/oxidative stress (Fig. 3g) and the survival of NeuN-positive neurons in the central region of hematoma (Fig. 3h). We also confirmed by Nissl staining that hematoma produced in the striatum expanded into the internal capsule in all cases and that the injury volume estimated by integration of injured areas of coronal sections was not different between AAV-GFP group and AAV-GFP-Nurr1 group (Supplementary Fig. 3a–c). In addition, ICH-induced defects of axonal transport, reflected by accumulation of diffused APP immunoreactivity associated with hematoma, were not affected by Nurr1 overexpression (Supplementary Fig. 3d and e).

### Nurr1 overexpression in the primary motor cortex recruits ret tyrosine kinase signaling

**Fig. 4:**
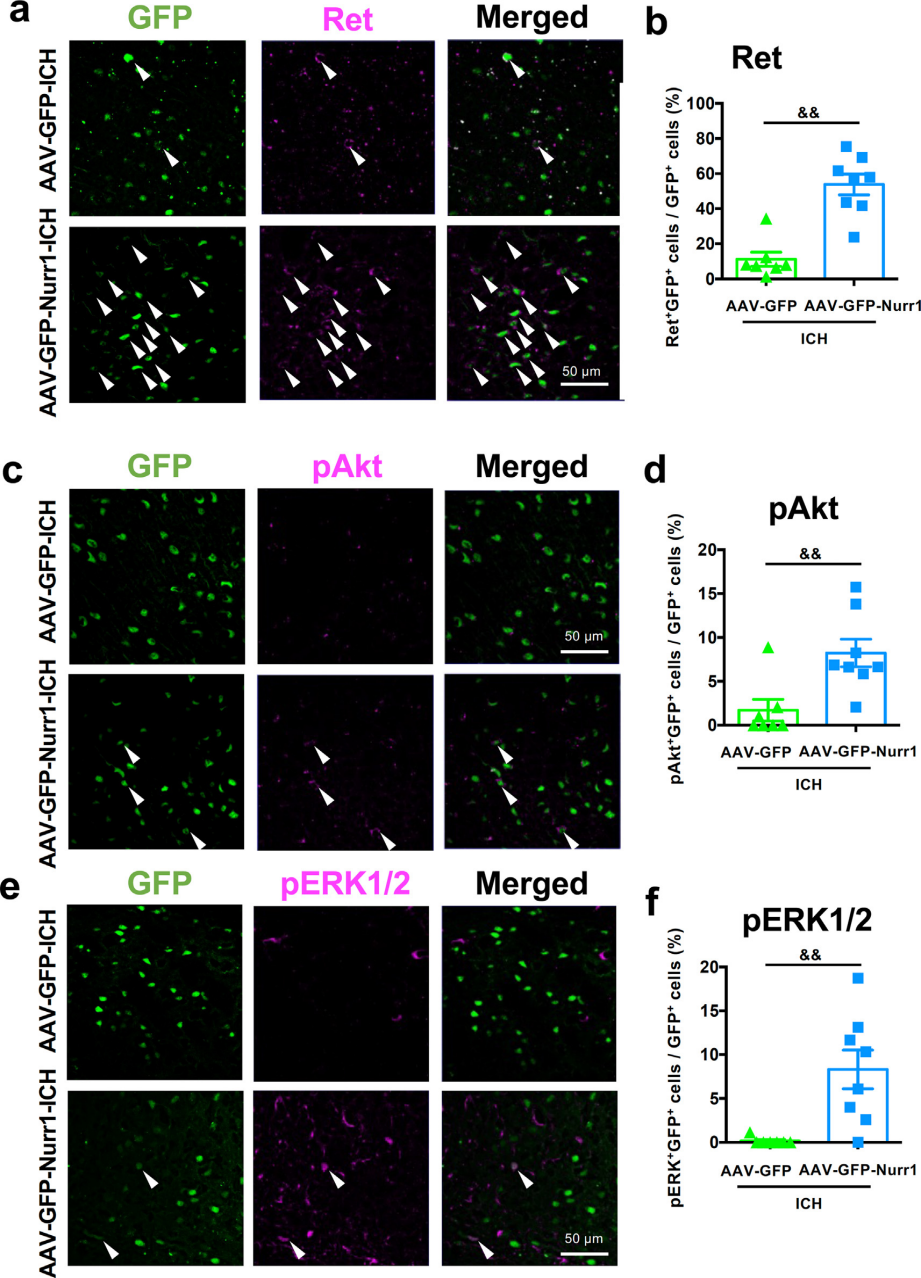
Effect of Nurr1 overexpression on Ret signaling in the cortex after ICH induction. **a** Representative pseudocolor images of GFP fluorescence and Ret immunofluorescence. Arrowheads indicate merged cells. **b** Quantitative results of the percentage of Ret/GFP-positive cells in GFP-positive cells. **c** Representative pseudocolor images of GFP fluorescence and phosphorylated Akt (pAkt) immunofluorescence. Arrowheads indicate doubly positive cells. **d** Quantitative results of the percentage of pAkt/GFP-positive cells in GFP-positive cells. **e** Representative pseudocolor images of GFP fluorescence and phosphorylated ERK1/2 (pERK) immunofluorescence. Arrowheads indicate doubly positive cells. **f** Quantitative results of the percentage of pERK/GFP-positive cells in GFP-positive cells. *$$ P* ​< ​0.01. Data were statistically analyzed by unpaired *t-*test (**b** and **d**) and Mann-Whitney test (**f**), respectively. Number of mice examined was 7 in AAV-GFP ​+ ​ICH group, and 8 in AAV-GFP-Nurr1 ​+ ​ICH group, respectively.

In the substantia nigra, Ret tyrosine kinase is known to maintain the survival of dopaminergic neurons [34,35]. Notably, Nurr1 has been reported to activate transcription of *Ret* gene and exert a neuroprotective effect in the substantial nigra [36]. We therefore examined the effect of Nurr1 overexpression on the expression of Ret in the cerebral cortex. Scattered expression of Ret was detected in the primary motor cortex of mice infected with AAV-GFP, and Ret-immunopositive signals were rarely colocalized with GFP fluorescence. On the other hand, Nurr1 overexpression by AAV-GFP-Nurr1 increased Ret expression, and there was a significant increase in the percentage of Ret-immunopositive cells within GFP-positive cell population (Fig. 4a and b).

Ret mediates intracellular signaling of glial cell line-derived neurotrophic factor (GDNF), with the aid of GDNF family receptor α1 (GFRα1) [37]. Signaling events downstream of Ret include phosphorylation of Akt and ERK1/2, both of which are implicated in neuronal survival and neurite outgrowth [[38], [39], [40], [41]]. Therefore, we next examined the effect of neuronal Nurr1 overexpression in the primary motor cortex on the levels of pAkt and pERK1/2. In the case of mice infected with AAV-GFP, only a small population of GFP-positive cells exhibited pAkt-immunoreactive signals, and virtually no pERK1/2-immunopositive cells were observed in GFP-positive cells. Nurr1 overexpression by infection with AAV-GFP-Nurr1 prominently increased the percentage of pAkt- and pERK1/2-immunopositive cells in GFP-expressing cells (Fig. 4c–f).

### Nurr1 ligands recruit GDNF-Ret signaling pathways in the primary motor cortex

**Fig. 5:**
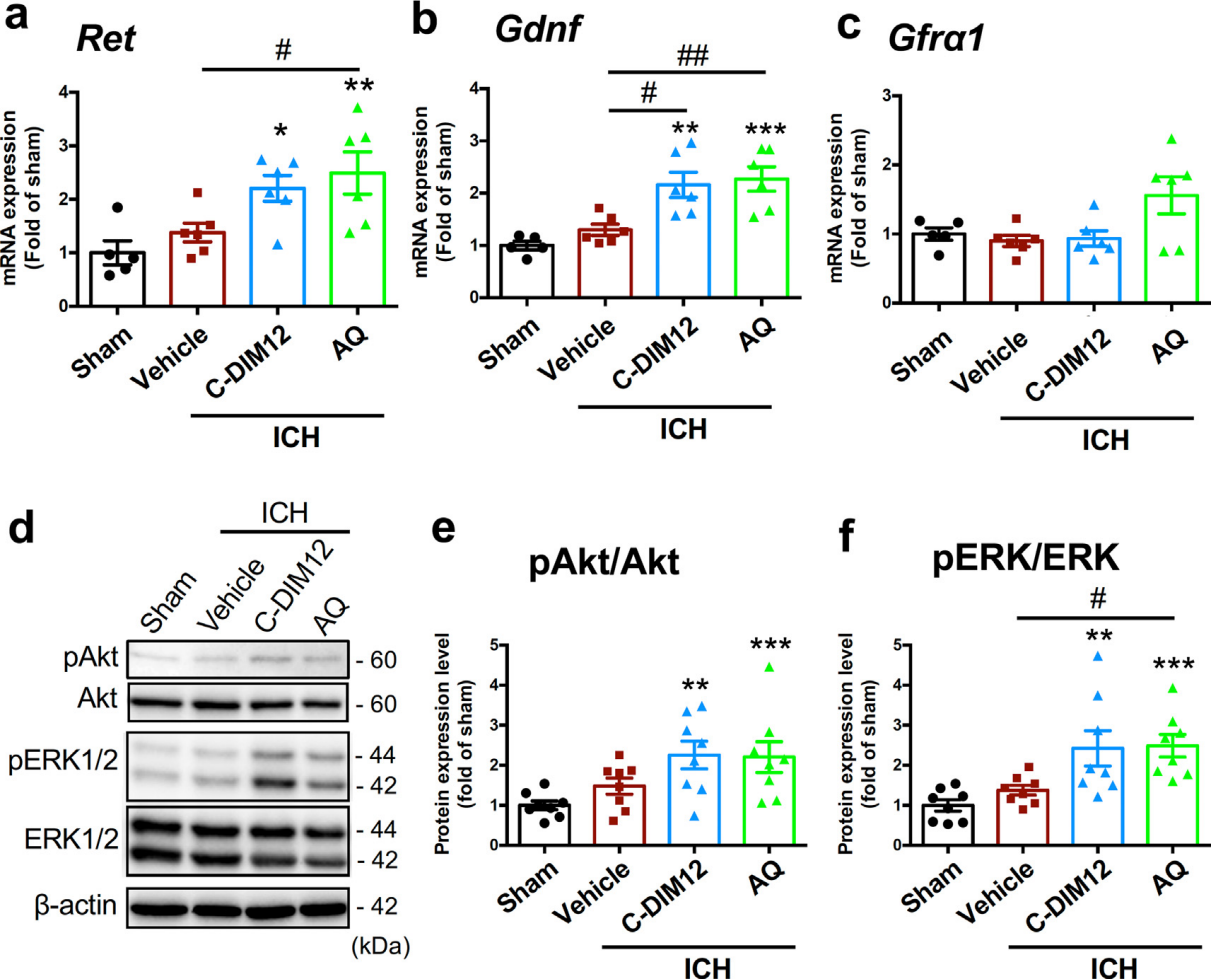
Effect of Nurr1 ligands on GDNF-Ret pathway in the cortex after ICH induction. **a-c** Quantitative results of mRNA expression levels of *Ret* (**a**), *Gdnf* (**b**) and *Gfrα1* (**c**) in cortical tissues obtained 1 ​d after sham operation or ICH induction. Number of mice examined was 5 in sham group, 6 in ICH ​+ ​vehicle group, 6 in ICH ​+ ​C-DIM12 group and 6 in ICH ​+ ​AQ group, respectively. **d** Representative blots of pAkt, Akt, pERK1/2, ERK1/2 and β-actin in cortical tissues obtained 3 ​d after sham operation or ICH induction. **e-f** Quantitative results of phosphorylation levels of Akt (**e**) and ERK1/2 (**f**). Number of mice examined was 8 in each group. C-DIM12 (50 ​mg/kg, p.o.) and AQ (40 ​mg/kg, i.p.) were administered daily, with the first administration given at 3 ​h after induction of ICH. *∗P* ​< ​0.05, *∗∗P* ​< ​0.01 and *∗∗∗P* ​< ​0.001 versus sham; *#P* ​< ​0.05 and *##P* ​< ​0.01. ANOVA results: for A, *F*_3, 19_ ​= ​6.18, *P* ​< ​0.01; for B, *F*_3, 19_ ​= ​10.87, *P* ​< ​0.001; for E, *F*_3, 28_ ​= ​4.55, *P* ​< ​0.05; for F, *F*_3, 28_ ​= ​7.288, *P* ​< ​0.01.

In the final set of experiments we examined the effect of Nurr1 ligands on GDNF/Ret-related signaling molecules in the cerebral cortex after ICH induction. In addition to transcriptional regulation of *Ret* gene, Nurr1 has also been reported to bind directly to the *Gdnf* gene promoter [42]. To verify whether Nurr1 ligands could regulate the expression of Ret and GDNF in the cerebral cortex, we performed RT-qPCR analysis on *Ret*, *Gdnf* and *Gfrα1* mRNAs. C-DIM12 (50 ​mg/kg) or AQ (40 ​mg/kg) was administered to mice at 3 ​h after induction of ICH, and the cortical tissues were obtained at 1 ​d after ICH induction. We found that ICH *per se* had no significant effect on the expression levels of *Ret*, *Gdnf* and *Gfrα1* mRNAs as compared to sham control. However, the expression levels of *Ret* and *Gdnf* mRNAs were significantly increased by single administration of C-DIM12 or AQ (Fig. 5a and b). These Nurr1 ligands had no effect on the level of *Gfrα1* mRNA (Fig. 5c).

We also examined whether signaling pathways downstream of GDNF/Ret were activated in the cerebral cortex by Nurr1 ligands. Similar to the case with the results of RT-qPCR experiments, ICH *per se* did not significantly affect the phosphorylated levels of Akt and ERK1/2 in the cerebral cortex after 3 ​d. In contrast, daily treatment with C-DIM12 or AQ significantly increased the levels of pAkt and pERK (Fig. 5d–f).

## Discussion

In our previous studies we have reported that several Nurr1 ligands such as C-DIM12 and AQ could alleviate neurological dysfunction in an experimental model of ICH in mice [[19], [20], [21]]. We have also shown that these Nurrl ligands could attenuate various pathogenic events associated with ICH. However, the relationship between the beneficial effect on neurological functions and the ameliorating effects on histopathological and biochemical events was unclear. Taking advantage of the properties of Nurr1 that posesses constitutive activity as nuclear transcription factor [15], we investigated the effect of Nurr1 overexpression on neurological outcome after ICH. We demonstrated here that Nurr1 overexpression in neurons of the primary motor cortex was sufficient to alleviate neurological deficits induced by ICH in the striatum.

The reason why we focused on the primary motor cortex was that pyramidal neurons in this brain region are the origin of CST, i.e., descending axonal projections that command activities of skeletal muscles via lower motor neurons in the spinal cord. Therefore, the integrity of CST is fundamental for executing normal locomotor activities. Indeed, ICH brings about severe motor dysfunction when hemorrhage generated in the striatum expands into adjacent IC that contains CST [6]. Moreover, various compounds with distinct pharmacological properties, such as leukotriene B_4_ receptor antagonist [10] and retinoic acid receptor agonists [43] as well as Nurr1 ligands [19,21], are all effective in alleviating ICH-induced neurological deficits and preventing ICH-induced degeneration of axonal structures in IC.

To achieve neuron-specific overexpression of Nurr1, we constructed AAV-GFP-Nurr1 that expressed GFP and Nurr1 under the control of hSynI promotor. AAV-GFP that expressed GFP only was used as control. In preliminary examinations *in vitro*, we confirmed expression of GFP by infection with AAV-GFP and expression of GFP and Nurr1 by infection of AAV-GFP-Nurr1, in cultured cerebellar neurons where endogenous Nurr1 protein expression is not detectable [44]. After confirming the validity of these AAV vectors, we injected them into the primary motor cortex of mice and successfully introduced neuronal expression of GFP and Nurr1, without accompanying any deleterious influences on body weight and sensorimotor functions of mice. After introduction of GFP/Nurr1 expression, we induced ICH that was initiated in the striatum and expanded into IC. GFP fluorescence could be used to identify axons as well as cell bodies of neurons infected with AAV vectors, and based on the shape of GFP fluorescence signals in IC, we found that Nurr1 overexpression in cortical neurons protected descending axonal projections from ICH-induced injury. This protective effect of Nurr1 overexpression was paralleled by better recovery of motor functions of mice after ICH, as shown by improved parameters in the beam-walking test. On the other hand, Nurr1 overexpression produced only modest effect on the performance of mice in the modified limb-placing test that assessed sensory as well as motor functions. These observations are consistent with the fact that ascending axonal projections for conducting sensory information to the primary sensory cortex also pass through IC and may undergo degeneration in response to ICH. That is, Nurr1 overexpression restricted in the primary motor cortex and descending axonal projections may be insufficient for ameliorating neurological functions that heavily rely on sensory information processing.

Notably, Nurr1 overexpression in the primary motor cortex produced no significant effect on various neuropathological events associated with hematoma, including activation of microglia/macrophages and astrocytes in the peri-hematoma region, increase in oxidative/nitrosative stress, decrease in surviving neurons and dysfunction of axonal transport within the hematoma. These results are different from previous findings obtained by systemic administration of Nurr1 ligands. For example, daily administration of C-DIM12 inhibited activation of microglia/macrophages, suppressed oxidative/nitrosative stress, prevented neuron loss and alleviated axonal transport dysfunction in the hematoma [19]. Daily administration of AQ inhibited activation of microglia/macrophages and astrocytes in the peri-hematoma region, and alleviated axonal transport dysfunction [19,20]. Taken together, these results suggest that the beneficial effect of Nurr1 overexpression under the present experimental conditions was specific to cortical neurons and further substantiate the proposal that protection of CST from ICH-induced injury is sufficient for amelioration of motor deficits.

Detailed mechanisms of protection of CST by Nurr1 overexpression remain to be determined, but one of the plausible mechanisms was recruitment of GDNF-Ret signaling pathway. In midbrain dopaminergic neurons, Nurr1 plays an important role in the maintenance of cell survival via transcription of several genes including Ret tyrosine kinase [45]. Ret is known to drive signaling by binding of GDNF-GFRα1 complex to regulate neuronal survival, neurite outgrowth and synapse formation [36]. We showed in the present study that Nurr1 overexpression indeed upregulated the expression of Ret proteins in neurons of the primary motor cortex. In addition, we found that administration of Nurr1 ligands C-DIM12 and AQ upregulated expression of mRNAs encoding GDNF as well as Ret in the cerebral cortex. Although Nurr1 has been reported to bind directly to the *Gdnf* promoter [41], to our knowledge, the present study is the first to demonstrate that pharmacological stimulation of Nurr1 increases *Gdnf* gene expression. We should note here that the endogenous expression level of Nurr1 in the primary motor cortex seems to be low (Fig. 1c–d) as compared to upregulated levels of Nurr1 expression in hematoma-associated glial cells [20], but modest and widespread expression of Nurr1 has been reported in mouse cerebral cortex [46]. As for components of signaling pathways downstream of GDNF-Ret, we showed that both Nurr1 overexpression and administration of Nurr1 ligands increased phosphorylation levels of Akt and ERK1/2. In this context, phosphorylation of Akt and ERK1/2 is implicated in GDNF-Ret signaling that mediates neuronal survival [[37], [38], [39], [40]]. Recruitment of GDNF/Ret/Akt/ERK signaling under the present experimental conditions provides additional line of evidence that Nurr1 activation in the motor cortex may contribute to the therapeutic effects of Nurr1 ligands on ICH. However, whether these signaling molecules indeed play an important role in maintaining the integrity of CST under ICH insults should be determined by further investigations. Further investigations, such as knockdown of endogenous Nurr1 and/or manipulation of relevant signaling pathways specifically in neurons of the primary motor cortex, may provide detailed mechanistic insights into the roles of Nurr1 and GDNF-Ret signaling.

In conclusion, AAV-mediated neuron-specific Nurr1 overexpression in the primary motor cortex protected CST against ICH-induced injury and improved motor dysfunction after ICH. These findings not only provided a clue to elucidate the mechanisms of the therapeutic actions of Nurr1 ligands on ICH, but also propose the primary motor cortex as the key brain region to develop effective drug therapies for ICH. Moreover, a recent study on postmortem brain tissues of multiple sclerosis patients demonstrated that Nurr1 expression in neurons of the primary motor cortex was upregulated in patients as compared to non-neurological controls. Interestingly, increased Nurr1 expression was positively associated with neuronal densities, suggesting that Nurr1 protects motor cortical neurons from multiple sclerosis-related injury [47]. These findings, combined with those of the present study, support the proposal that Nurr1 in the primary motor cortex may serve as an important therapeutic target for several distinct types of neurological disorders.

## Author contributions

K.K., T.S. and H.K. designed the study. K.K., K.M., K.U. and Y.H. performed the experiments. A.K. and H.H. constructed AAV vectors. S.K. synthesized and provided a reagent. K.K., K.M., K.U. and Y.H. analyzed the experimental data. K.M., K.U., Y.H., N.H.-I., Y.K. and T.S. made suggestions to the improvement of the study. K.K. N.H.-I., Y.K., T.S. and H.K. wrote manuscript draft. The authors read and approved the final manuscript.

## Declaration of competing interest

The authors have no relevant financial or non-financial interests to disclose.
