# Supplementary material for: Nurr1 overexpression in the primary motor cortex alleviates motor dysfunction induced by intracerebral hemorrhage in the striatum in mice
Source: Neurotherapeutics. 2024 May 3;21(4):e00370. doi: 10.1016/j.neurot.2024.e00370 (PMC11305294; doi:10.1016/j.neurot.2024.e00370)
Supplement: Multimedia component 1 [file mmc1.pdf]

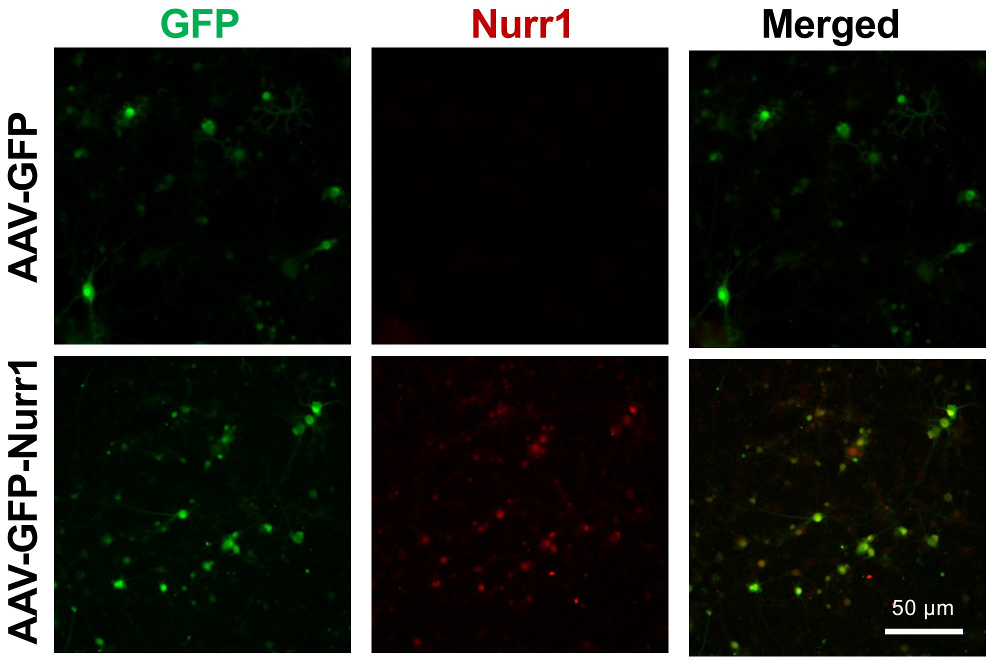

Supplementary Fig. 1 Confirmation of Nurr1 gene introduction in neuronal cells by AAV vector. Primary cultures of cerebellar cells dissociated from rat embryos were transfected with AAV-GFP or AAV-GFP-Nurr1. GFP fluorescence and Nurr1 immunofluorescence were observed at 14 d after infection of AAVs.

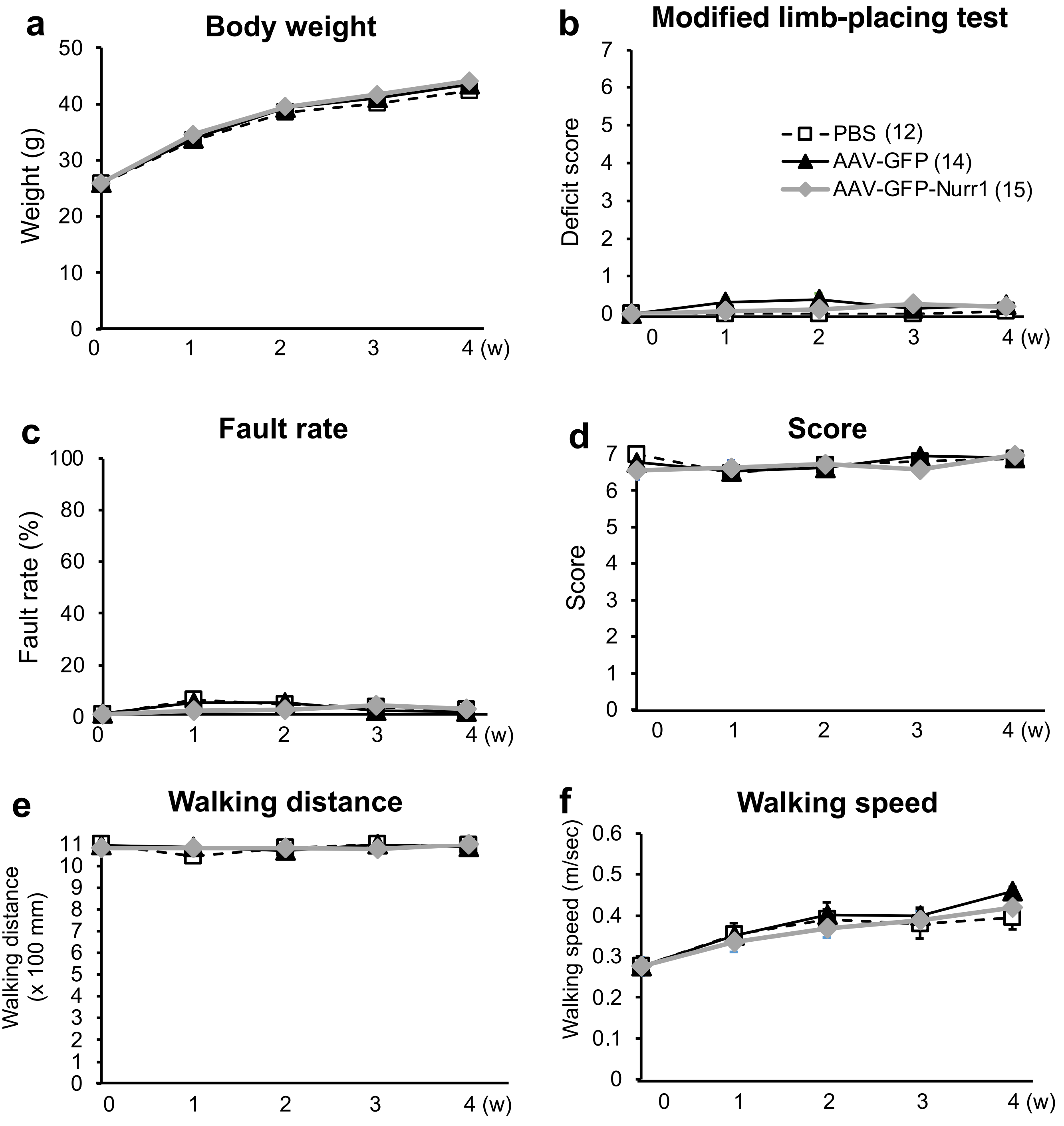

Supplementary Fig. 2 Body weight and neurological parameters of mice after injection of PBS or AAV vectors into the primary motor cortex. Time-dependent changes in body weight (**a**), deficit score in the modified limb-placing test (**b**), fault rate (**c**), performance score (**d**), walking distance (**e**) and walking speed (**f**) in the beam-walking test were monitored for 4 weeks after injection. No significant differences between groups were observed by two-way repeated measure ANOVA concerning body weight (interactions, $F_{8, 152} = 0.5842$, $P = 0.7898$; time, $F_{4, 152} = 921.8$, $P < 0.001$; treatment, $F_{2, 38} = 0.8909$, $P = 0.4187$), the modified limb-placing test (interactions, $F_{8, 148} = 1.462$, $P = 0.1758$; time, $F_{4, 148} = 2.296$, $P = 0.0619$; treatment, $F_{2, 37} = 2.929$, $P = 0.0659$), the fault rate (interactions, $F_{8, 148} = 3.132$, $P < 0.01$; time, $F_{4, 148} = 12.54$, $P < 0.001$; treatment, $F_{2, 37} = 1.065$, $P = 0.3551$), the performance score (interactions, $F_{8, 148} = 1.177$, $P = 0.3164$; time, $F_{4, 148} = 3.054$, $P = 0.0188$; treatment, $F_{2, 37} = 0.1724$, $P = 0.8423$), the walking distance (interactions, $F_{8, 148} = 1.543$, $P = 0.1472$; time, $F_{4, 148} = 2.639$, $P = 0.9066$; treatment, $F_{2, 37} = 0.09828$, $P = 0.9066$) and the walking speed (interactions, $F_{8, 148} = 0.7803$, $P = 0.6207$; time, $F_{4, 148} = 34.09$, $P < 0.001$; treatment, $F_{2, 37} = 0.3284$, $P = 0.7222$). Number of animals examined was 12 in PBS group, 14 in AAV-GFP group and 15 in AAV-GFP-Nurr1 group, respectively.

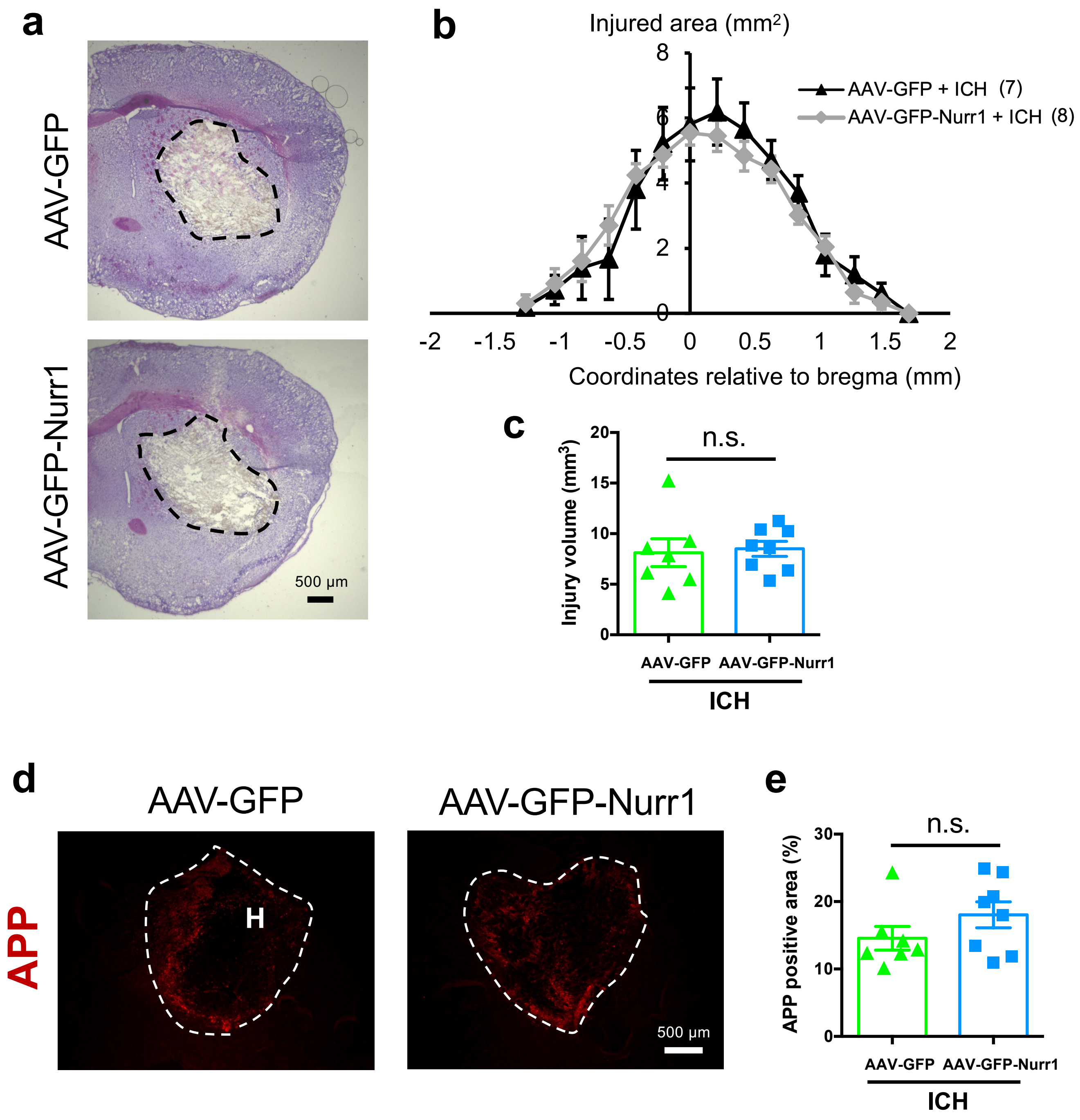

Supplementary Fig. 3 Effect of Nurr1 overexpression on injury volume and axonal transport after ICH induction. **a** Representative images of Nissl-stained coronal sections obtained at 3 days after ICH. **b-c** Quantitative results of injury area (**b**) and injury volume (**c**) at 3 d after ICH induction. No significant differences between groups were observed by two-way repeated measure ANOVA concerning injury area (interactions, $F_{14,\,182} = 0.5599$, $P = 0.8932$; coordinate, $F_{14,\,182} = 40.08$, $P < 0.001$; treatment, $F_{1,\,13} = 0.03$, $P = 0.8653$). n.s. = not significant. **d** Representative images of amyloid precursor protein (APP) obtained at 3 d after ICH. H; hematoma. **e** Quantitative results of APP-positive area. Number of animals examined was 7 in AAV-GFP + ICH group and 8 in AAV-GFP-Nurr1 + ICH group, respectively.
